# News and Coming Events

**Published:** 1908-07-18

**Authors:** 


### News and Coming Events.

Mr. W. S. Nowell, M.A., M.R.C.S., L.D.S., has been appointed dental surgeon to the Middlesex Hospital.

St. Mary's Hospital has received an anonymous donation of £5,000 in relief of its great financial necessities.

Madame Melba has sent to the London Hospital a cheque for £2,000, being the proceeds of the matinée at Covent Garden which she organised on behalf of the institution.

Louis Francis Knuthsen, M.D.Edin., has been appointed Assistant Physician to St. John's Hospital for Diseases of the Skin, Leicester Square, W.C.

The annual meeting of the Medico-Psychological Association will be held under the presidency of Dr. Charles Mercier at 11 Chandos Street on July 23 and 24.

In the announcement of the recent Birthday Honours in our issue of July 4, we omitted to record the knighthood of Dr. Peter O'Connell, surgeon to the Mater Infirmorum Hospital and High Sheriff of Belfast.

A Site has been chosen at Worthing for the convalescent home which is to be erected and endowed in connection with the Royal Surrey County Hospital at Guildford, by the generous gift for that purpose of £10,000 from Mr. George Prentiss Shepherd, of Keymer, Sussex. On its completion Surrey will have convalescent homes at Worthing, Bognor, and Seaford, all three of which are county institutions.

An official attempt is being made by the German Government to suppress trichinosis. As rats are known to be the carriers of trichinosis, steps are to be taken in the first place towards the extermination of rats, and the campaign is to be undertaken by extensive applications of ratin. Four hundred and sixty farms are to be treated, involving a probable area of 100,000 acres.

The Council of the Royal College of Surgeons of England has decided to recognise the *Dreadnought* Hospital, Greenwich, for the additional year of surgical hospital practice required in the case of candidates for the Fellowship who are not members of the College; and to place Laval University, Quebec, McGill University, Montreal, and the Italian Royal Universities on the list of Universities the degrees of which are accepted as qualifying for admission to the Final Examination for the diploma of Fellow.

The Duchess of Buccleuch presided at the annual meeting of the Westminster Hospital Ladies' Association. The excellent work which the Association is doing on behalf of the hospital was described in the annual report, which stated that thirty-eight members and associates had been added during the year. It was desired to increase the junior branch to at least six hundred members, which would enable the children to support a cot entirely themselves. During the year the Association had provided £30 for the support of the cot in Holland Ward, and various contributions had been made to the funds of the institution. There was a sum of £151 in hand towards the amount required—namely, £600—for the permanent endowment of a cot. The report was adopted, and the appointment of Mr. Charles Geake as hon. secretary was confirmed.

The total received to date at the Mansion House for the Metropolitan Hospital Sunday Fund is about £33,000.

The Royal Waterloo Hospital for Children and Women has received the munificent gift of £13,105 (the interest on which only can be used) from a donor who wishes to remain anonymous. The Board wish to express their most hearty thanks for this splendid donation, which will considerably lighten their responsibilities at a time when funds are so very urgently needed by this poor, but most deserving, charity.

At a meeting on July 9 at Grosvenor House, presided over by the Duke of Marlborough, it was decided that an appeal should be made to the public for £25,000 on behalf of the Royal National Orthopædic Hospital, with which is incorporated the City Orthopædic Hospital. The King and Queen are patrons of the hospital, and His Majesty has recently expressed approval of the work which is being done. Contributions should be addressed to the secretary of the hospital, in Great Portland Street.

In reply to a communication from Mr. G. W. F. Robbins, the Secretary of the National Anti-Vivisection Hospital, Battersea, Sir Edmund Hay Currie, Secretary of the Metropolitan Sunday Fund, stated that the committee of distribution did not see its way to make an award to the hospital, but would be glad to receive a deputation if there had been any alteration in the constitution of the hospital. Sir Edmund was informed by the Chairman of the Board of Management of the hospital that there could be no change in regard to the utter banishment of vivisection, and the chairman added that in the opinion of his board the refusal of a grant was inconsistent with the equitable administration of a charitable fund to which the general public had largely contributed.

A Reuter telegram from Brisbane, dated July 9, announces the death of Sir Thomas Naghten Fitzgerald, the famous surgeon. Sir Thomas Fitzgerald, who was born in 1838, was consulting surgeon to the Melbourne, St. Vincent, Austin, and Queen Victoria Hospitals. Sir Thomas was a Fellow of the Royal College of Surgeons, Ireland, and of the Royal College of Surgeons, Edinburgh. In 1889 he was President of the Intercolonial Medical Congress of Australasia, and he was twice—in 1884 and 1890—President of the Medical Society of Victoria. During the war in South Africa Sir Thomas Fitzgerald acted as consulting surgeon to the Imperial forces, being mentioned in despatches and receiving a C.B.

The Liverpool School of Tropical Medicine have two men specially studying trypanosomiasis in North-Western Rhodesia at the present moment. They have recently published some reports in the Annals of Tropical Medicine and Hygiene. Their most important conclusions are that there are several species of trypanosomes implicated in the trypanosomiasis of animals in Rhodesia and that other biting flies than the tsetse fly may transmit the parasite from animal to animal. These points are really of very great importance, and confirmation of them by the Royal Society's Commission would be interesting. Other proofs are still required on other manifestations of the disease, but with so many commissions constantly going out to Africa the doubtful points should soon be cleared up.

At the quarterly meeting of the Council of the Royal College of Surgeons, held on July 9, Mr. Henry Morris was re-elected President, and Sir W. Watson Cheyne, C.B., F.R.S., and Mr. A. Pearce Gould were elected Vice-Presidents for the ensuing year.

At the same meeting the Council elected the following professors and lecturers for 1908-1909. Hunterian Professors, Messrs. Arthur Keith, S. G. Shattock, William Wright, and C. W. Rowntree. Arris and Gale Lecturer, Dr. G. Elliot Smith. Erasmus Wilson Lecturer, Mr. S. G. Shattock.

Mr. Archibald John Thomas Francis Aitchison, of Bournemouth, barrister-at-law, has bequeathed, on the death of his wife, £5,000 to the East London Hospital for Children conditional upon the Committee maintaining in good repair and condition the grave of his late wife, and also his father's vault. Failing the observance of these conditions the legacy is to revert to the Alexandra Hospital for Children with Hip Disease, Queen Square, Bloomsbury.

In aid of the League of Mercy, a Hungarian Village Fête is being organised at the Exhibition, Earl's Court, on Tuesday, July 21, from 3 to 11 p.m. The scene of the fête will be the Hungarian Village; a number of distinguished Presidents of the League of Mercy will preside at the different picturesque cottages, and the profits of the quaint and rustic wares sold at the Exhibition on that day will benefit the League of Mercy. A series of concerts and national dances will be given on the occasion, and several distinguished Hungarian artistes will come over expressly for the purpose.

Lord Howard de Walden, chairman of the hospital, presided at the anniversary festival dinner of the Metropolitan Hospital, Kingsland Road, held on July 13 at the Hôtel Métropole. The chairman proposed the toast "Prosperity to the Hospital." Mr. Leopold Rothschild, one of the treasurers, in responding, spoke of the good work which the hospital was doing in a poor and populous district. They owed £6,000 to tradespeople, which had to be paid, and they also wanted to provide better accommodation for the nurses. The Hon. Claude Hay, M.P., gave the toast of "The Committee of Management," and Mr. C. J. Thomas, chairman of the Committee, responded. During the evening subscriptions to the amount of £3,501 1s. were announced, the chairman heading the list with 300 guineas.

On July 10 Lord Crewe visited the London Hospital to distribute the prizes won by medical students and nursing probationers, and afterwards to unveil the statue of Queen Alexandra which has been erected in the grounds behind the hospital. Lord Crewe said his was the high honour of unveiling the first statue of Queen Alexandra erected in this country. Looking back through the records of the consorts of our kings, even to the days of Henry the First's "Good Queen Maud," he did not think that one had been so completely enthroned in the hearts of the nation as Queen Alexandra. When presenting the prizes and certificates the Colonial Secretary remarked that there are few great prizes in the medical profession, but that doctors have one great reward denied to politicians, for they continually see the direct results of their work, whereas the procession of events by which political improvements work is nearly always extremely slow. Lord Crewe repudiated the charge that medical training tends to make a man callous; the standard of humanity among medical men is, he said, higher than the average.

Dr. Thomas G. Stevens has been elected a physician to out-patients of Queen Charlotte's Lying-in Hospital.

Queen Charlotte's Hospital has received a donation of £500 from Viscount Portman for the Nurses' Home Extension Fund.

Lord Farquhar and Sir Richard B. Martin, Bart., treasurers of the Royal National Orthopædic Hospital, have received a donation of 100 guineas from Mrs. James Packe, and £50 from Cecil H. Oliverson, Esq., towards the Rebuilding Fund of that Hospital.

In the thirteenth list of units of the Territorial Force reported as having attested at least 30 per cent. of the authorised establishment and received the recognition of the Army Council are included the following medical units: The Second Western General Hospital, First Southern General Hospital, Third Scottish General Hospital, Fourth Scottish General Hospital, and First Western General Hospital.

The Lord Mayor's dinner on July 6 on behalf of the London Hospital Quinquennial appeal was a great success from every point of view. The number present was 264. Among the more important speeches were those of the Lord Mayor, Lord Rothschild, and the Hon. Sidney Holland. In addition to the £5,000 given to the Lord Mayor before the dinner, £7,000 was subscribed in the room. This is all the more encouraging, since most of those present had already given to the Fund.

At the final meeting of the Committee for the concert at Albert Hall given recently to workers of the League of Mercy, the hon. treasurer, Mr. E. W. Wallington, submitted to Princess Victoria of Schleswig-Holstein, who presided, and to the committee, a statement of the accounts, showing a surplus of £728 19s. 2d., being the result of the sale of tickets after 5,000 invitations had been issued and all expenses had been paid. It was decided to allot £200 to the general purpose fund, £50 to the Queen's Nurses' Fund, £100 to the Reginald Lund Memorial Fund, and £378 19s. 2d. to King Edward's Hospital Fund, through the hon. treasurer of the League of Mercy.

The Army Council has notified the County Associations that consideration has been given to a system whereby the Territorial Medical Service and the Ambulance Department of the Order of St. John of Jerusalem may be brought into relation, so that, while each organisation shall maintain its own individuality, mutual aid and support may be rendered by one to the other. Many thousands of persons are annually trained by the Order; but while a large number of these pass into the St. John Ambulance Brigade, many others sever their connection with the organisation. On the other hand, soldiers of the Territorial Medical Service, on completion of their time, will not, under existing conditions, have opportunities of passing into any of the St. John organisations, in which the conditions of service and the liabilities are less stringent than in the Territorial Force, and in which they might continue to place at the disposal of the country the special training and knowledge acquired in the military service. After conference with a special committee of the Order of St. John, proposals have been submitted to the County Associations by which it is believed that both the Territorial Medical Service and the St. John Ambulance Brigade may be strengthened.

